# Commentary: Altered learning under uncertainty in unmedicated mood and anxiety disorders

**DOI:** 10.3389/fnhum.2020.561770

**Published:** 2020-11-13

**Authors:** Motofumi Sumiya, Kentaro Katahira

**Affiliations:** ^1^Department of Cognitive and Psychological Sciences, Graduate School of Informatics, Nagoya University, Aichi, Japan; ^2^Japan Society for the Promotion of Science, Tokyo, Japan

**Keywords:** hierarchical Bayesian estimation, shrinkage, computational psychiatry, computational modeling, reinforcement learning (RL)

Understanding how anxious and depressed individuals process information is a central topic in the field of psychiatry. In this regard, Aylward et al. (2019) utilized computational models of learning to better understand and describe how anxious and/or depressed individuals behave from moment to moment when faced with uncertain situations. Participants performed decision-making tasks characterized by fluctuating rewards and punishment. The authors fit computational models to the collected data from participants in the anxiety and healthy groups using the hierarchical Bayesian estimation with two levels of priors, at individual and group levels, where they set the group prior separately for each group. The three parameters of the winning model (punishment learning rate, lapse parameter, and decay rate) were higher in the symptomatic group than in the healthy group. In short, the authors found that anxious individuals quickly learned about negative phenomena but not about positive phenomena. Notwithstanding, we believe we have identified two methodological issues regarding the statistical analysis of the cited study, *shrinkage* and a “two-step approach.”

Visual inspection of parameter estimates (Figure 1A in the present article and Figure 2A in the original article) indicates that the punishment learning rate clustered at the higher value near its maximum (i.e., 1), suggesting that the decision-making of most participants in the symptomatic group depended solely on punishment outcomes in the immediate past. At first glance, this result appears to be too extreme although it could be a genuine reflection of anxious individuals' characteristics—overreacting when exposed to immediate punishments. However, we suggest that the original results might suffer from statistical bias caused by a property of the hierarchical Bayesian estimation procedure called *shrinkage*.

**Figure 1:**
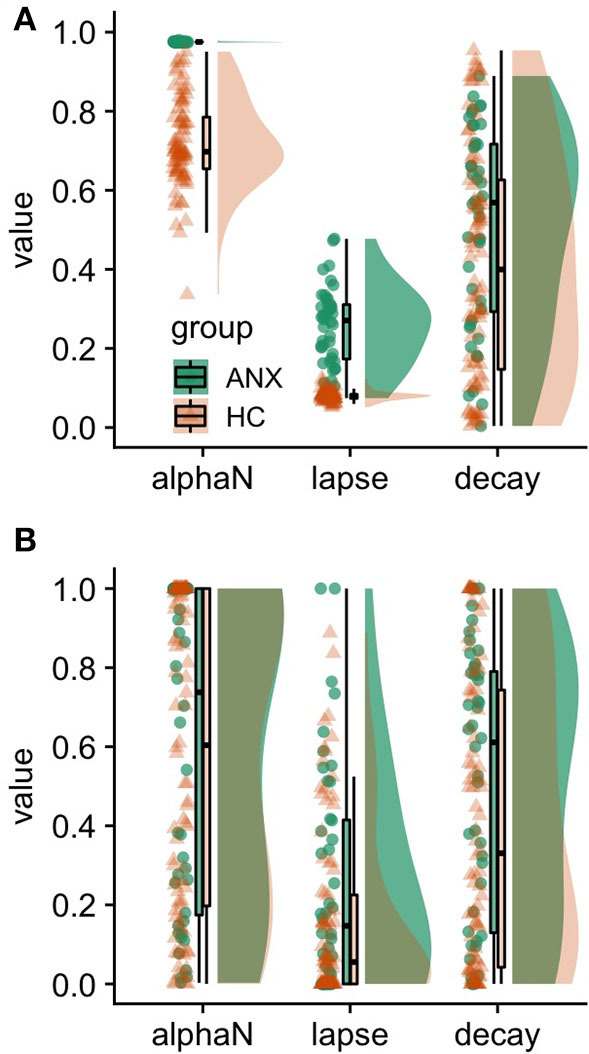
Estimated parameters of the winning (‘bandit4arm_lapse_decay’) model. **(A)** Hierarchical Bayesian parameter estimation, **(B)** maximum likelihood estimation. alphaN, Punishment learning rate; ANX, anxiety/symptomatic/experimental group; HC, healthy control group. Lapse parameter, noisiness of decision-making; decay rate, the propensity to forget the previous values of unchosen options. We found larger distributions for the punishment learning rate in the anxiety group, which is comparable to those in the healthy group **(B)**. These results indicate that a strong shrinkage occurred in the estimates of the punishment learning rate in the anxiety group in this data set **(A)**.

Shrinkage is a notable property of hierarchical models, in that less reliable estimates are more strongly biased toward the group mean (Efron and Morris, 1977). While the shrinkage property may improve the estimation of individual parameters (Ahn et al., 2014; Katahira, 2016), it may also lead to an underestimation of group-level variances (individual differences). To examine whether the small variances in Aylward et al.'s results were due to strong shrinkage, we conducted a maximum likelihood estimation with the same dataset since it does not have this shrinkage characteristic and it provides unbiased, albeit noisy, estimates for each participant. We found larger distributions for the punishment learning rate among the anxiety group, which were comparable to those for the healthy group (Figure 1B). These results indicated that a strong shrinkage occurred in the estimates for the punishment learning rate in the anxiety group.

In the original study, both groups were statistically compared regarding their group-level means for each parameter, rather than regarding individual-level estimates for each parameter. Thus, the shrinkage of estimates at the individual-level might not have directly influenced the results. However, too much shrinkage indicates that the variance of group-level distribution might have become improperly small (Supplementary Figure 1). This would also lead to the smaller variance of the posterior group-level mean distribution for the anxiety group (Supplementary Figure 2), which might inflate false-positive rates. A previous study has shown that improperly small population variance is often obtained when the analyzed data do not provide reliable information regarding the variances in study population distributions (Gelman et al., 2013). One potential source for this unreliable information refers to the interdependencies between model parameters (Scheibehenne and Pachur, 2015). These interdependencies make different parameter combinations equally probable, so the reliability of each parameter is diminished. To examine the influence of the interdependency of model parameters on Aylward et al.'s results, we calculated the correlation coefficient of the posterior distribution for the free parameters in the winning model among the symptomatic group. Indeed, we found that the punishment learning rate, which showed strong shrinkage, correlated with other parameters, including the decay rate (Supplementary Figure 3). Even though the distribution of the punishment learning rate of the second winning model (without decay rate) estimated maximum likelihood estimation (Supplementary Figure 4) is comparable to the one of the winning model (Figure 1B), the parameter on the second winning model estimated with hierarchical Bayesian parameter estimation did not seem to show strong shrinkage (Supplementary Figure 4A in this manuscript and Figure 2D in Aylward et al.), like the one with the winning model (Figure 1A). Therefore, interdependent correlations between punishment learning rate and decay rate in the winning model might have caused the observed strong shrinkage.

In addition to the between-group comparison regarding estimated parameters, to investigate the continuous relationship between symptom scores and model parameters, the original authors submitted the point parameter estimates obtained from individual participants into correlational statistical tests. However, this “two-step approach” (participant-level point estimates acquired by a hierarchical Bayesian estimation—that is independently applied to each group—being subsequently used in a frequentist test) has been criticized in the literature because it biases the results toward an alternative hypothesis (Boehm et al., 2018). This happens because the underestimated group-level variance leads to overestimated correlation coefficients, thereby causing Type I error rates to be inflated. Thus, the results shown in Figure 4 of the original manuscript should be interpreted with caution.

The use of a hierarchical Bayesian approach in computational modeling has been enhanced by the development of open-source software (e.g., hBayesDM; Ahn et al., 2017, which was used by Aylward et al.). Although this convenient and useful software may contribute magnificently to the development of research in the fields of psychology and psychiatry, an adequate understanding of their underlying mechanisms is required to ensure appropriate use. For example, the shrinkage degree often depends on the choice of the prior distribution for population distribution variances (Gelman, 2006). Although properties of the prior distribution used in Aylward et al. (e.g., the Cauchy distribution) have yet to be studied, there seems to be a high probability for strong shrinkage to occur if posterior distributions are near the edge of the original parameter range (around one). We believe that further theoretical consideration about the influences of the prior and the model structure is needed to explore the proper use of hierarchical Bayesian modeling in computational psychiatry.

## Code availability

All codes used in the analysis are available on the Open Science Framework (https://osf.io/rx8hz/).

## Data Availability Statement

Datasets used in this study are reuse of open datasets on the Open Science Framework (https://doi.org/10.17605/OSF.IO/UB6J7).

## Author Contributions

MS and KK provided theoretical input and designed this study. MS analyzed the data. MS and KK wrote the manuscript. Both authors contributed to the article and approved the submitted version.

## Conflict of Interest

The authors declare that the research was conducted in the absence of any commercial or financial relationships that could be construed as a potential conflict of interest.
