# Supplementary material for: Commentary: Altered learning under uncertainty in unmedicated mood and anxiety disorders
Source: Front Hum Neurosci. 2020 Nov 13;14:561770. doi: 10.3389/fnhum.2020.561770 (PMC7691592; doi:10.3389/fnhum.2020.561770)
Supplement: Supplementary file 1 [file Table_1.docx]

**Supplementary Information**

**Commentary: Altered learning under uncertainty in unmedicated mood and anxiety disorders**

Motofumi Sumiya^1,2,^*, Kentaro Katahira^1,^*

^1^ Department of Cognitive and Psychological Sciences, Graduate School of Informatics, Nagoya University, Aichi, Japan

^2^ Japan Society for the Promotion of Science, Tokyo, Japan

*Correspondence should be addressed to Motofumi Sumiya (motofumisumiya@gmail.com) and Kentaro Katahira (katahira.kentaro@b.mbox.nagoya-u.ac.jp)

**This file includes:**

4 Supplemental Figures


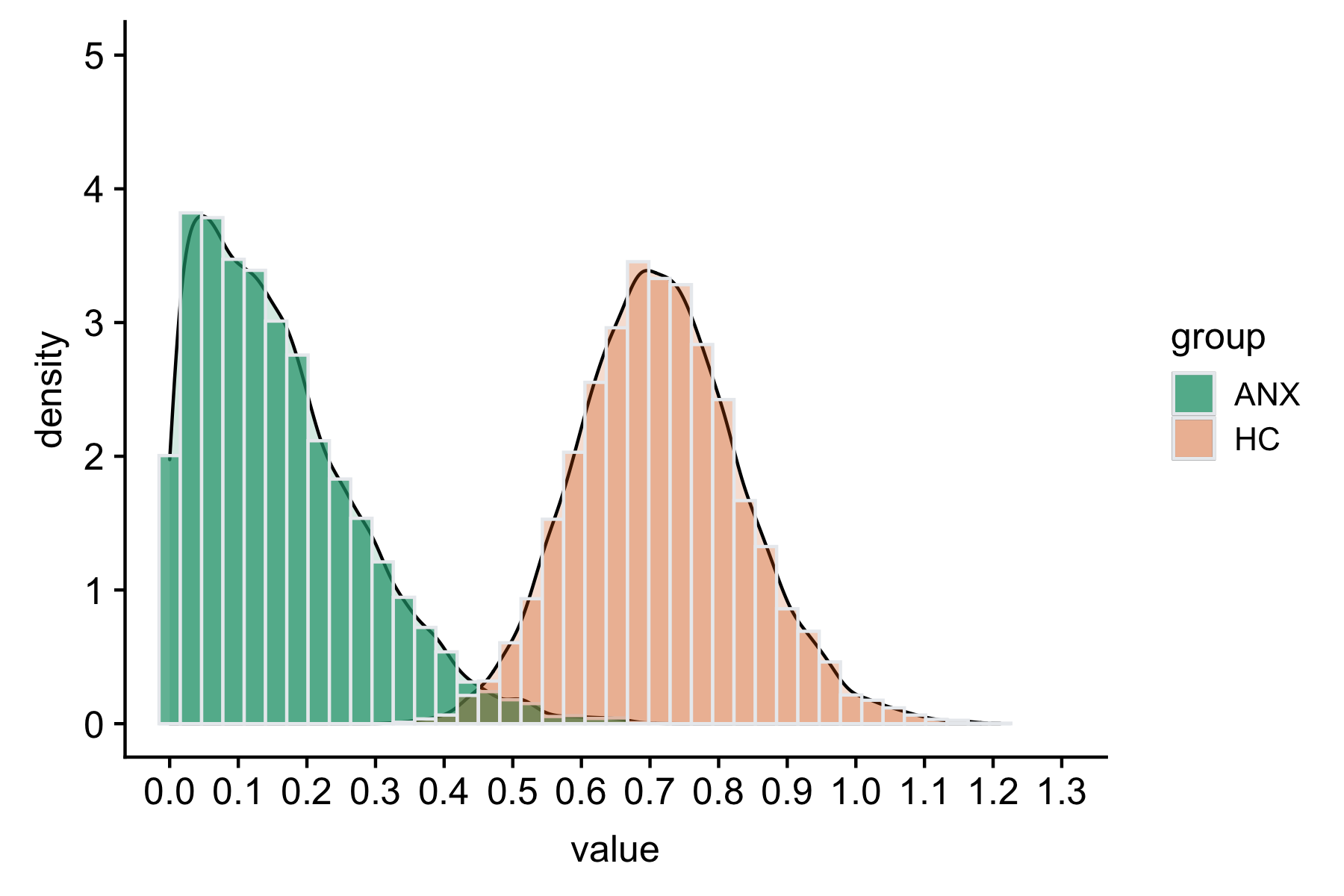


S1 Fig. Posterior distribution of variance of group-level distribution for punishment learning rate. ANX: anxiety group, HC: healthy control group.


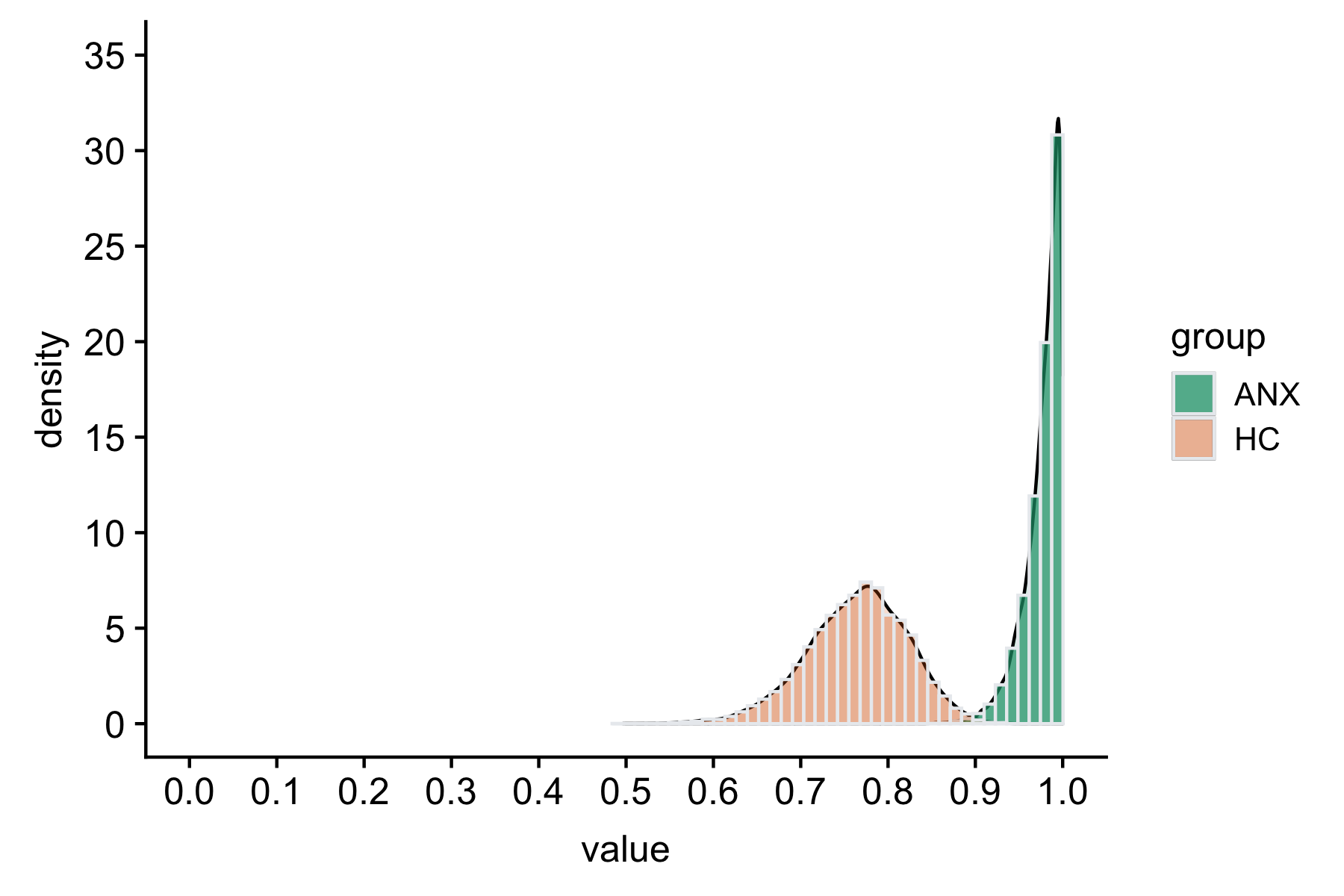


S2 Fig. Posterior distribution of means of group-level distribution for punishment learning rate. ANX: anxiety group, HC: healthy control group


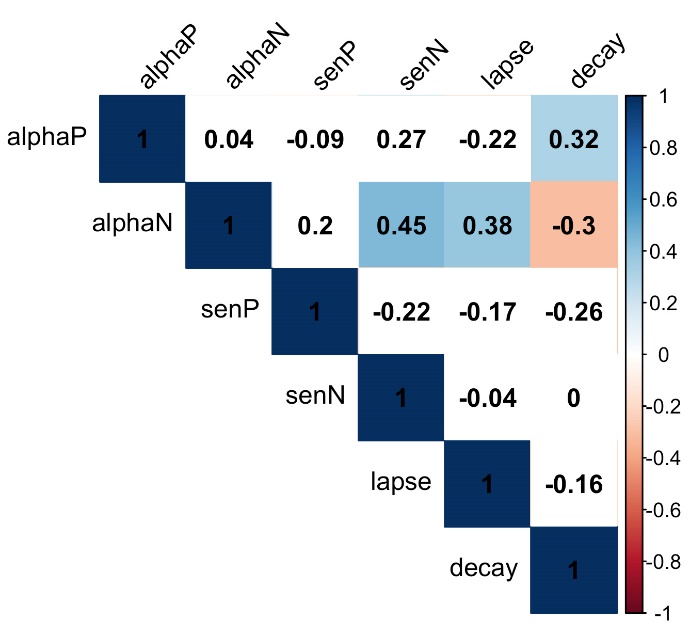


S3 Fig. Correlation matrix for posterior parameters of the bandit4arm_lapse_decay model for the anxious group. Numbers within the square refer to correlation coefficients. Coloured squares (p < 0.05). “alphaP” represents reward learning rate; “alphaN” represents punishment learning rate; this categorization extended to sensitivity (senP and senN).


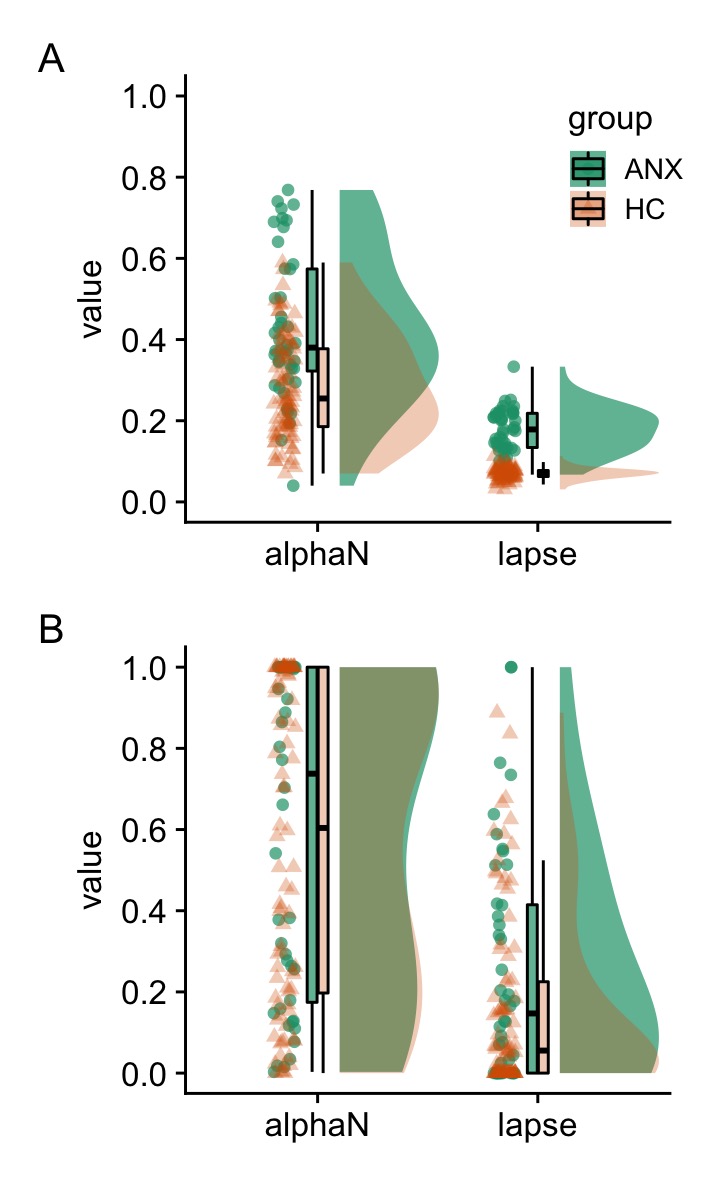


S4 Fig. Estimated parameters of the winning (‘bandit4arm_lapse’) model. A) hierarchical Bayesian parameter estimation, B) maximum likelihood estimation. alphaN: Punishment learning rate, ANX: anxiety/symptomatic/experimental group, HC: healthy control group. Lapse parameter: noisiness of decision-making.
